# Exploring the Intersection Between Health Professionals’ Learning and eHealth Data: Protocol for a Comprehensive Research Program in Practice Analytics in Health Care

**DOI:** 10.2196/27984

**Published:** 2021-12-09

**Authors:** Anna Janssen, Stella Talic, Dragan Gasevic, Judy Kay, Tim Shaw

**Affiliations:** 1 Faculty of Medicine and Health The University of Sydney Sydney Australia; 2 School of Public Health and Preventative Medicine Monash University Melbourne Australia; 3 Faculty of Information Technology Monash University Melbourne Australia; 4 Faculty of Engineering The University of Sydney Sydney Australia; 5 Digital Health Cooperative Research Centre Sydney Australia

**Keywords:** digital health, health informatics, practice analytics in health care, health professions education, continuing professional development

## Abstract

**Background:**

There is an increasing amount of electronic data sitting within the health system. These data have untapped potential to improve clinical practice if extracted efficiently and harnessed to change the behavior of health professionals. Furthermore, there is an increasing expectation from the government and peak bodies that both individual health professionals and health care organizations will use electronic data for a range of applications, including improving health service delivery and informing clinical practice and professional accreditation.

**Objective:**

The aim of this research program is to make eHealth data captured within tertiary health care organizations more actionable to health professionals for use in practice reflection, professional development, and other quality improvement activities.

**Methods:**

A multidisciplinary approach was used to connect academic experts from core disciplines of health and medicine, education and learning sciences, and engineering and information communication technology with government and health service partners to identify key problems preventing the health care industry from using electronic data to support health professional learning. This multidisciplinary approach was used to design a large-scale research program to solve the problem of making eHealth data more accessible to health professionals for practice reflection. The program will be delivered over 5 years by doctoral candidates undertaking research projects with discrete aims that run in parallel to achieving this program’s objectives.

**Results:**

The process used to develop the research program identified 7 doctoral research projects to answer the program objectives, split across 3 streams.

**Conclusions:**

This research program has the potential to successfully unpack electronic data siloed within clinical sites and enable health professionals to use them to reflect on their practice and deliver informed and improved care. The program will contribute to current practices by fostering stronger connections between industry and academia, interlinking doctoral research projects to solve complex problems, and creating new knowledge for clinical sites on how data can be used to understand and improve performance. Furthermore, the program aims to affect policy by developing insights on how professional development programs may be strengthened to enhance their alignment with clinical practice. The key contributions of this paper include the introduction of a new conceptualized research program, Practice Analytics in Health care, by describing the foundational academic disciplines that the program is formed of and presenting scientific methods for its design and development.

**International Registered Report Identifier (IRRID):**

PRR1-10.2196/27984

## Introduction

### Background

Emerging digital technologies for collecting and using eHealth data have the potential to make data more accessible to individual clinicians, clinical teams, organizations, and the general public. The increasing accessibility of eHealth data provides opportunities for their use in a wide range of applications, including quality improvement activities [1], improving clinical processes [2], and facilitating new approaches to clinical research [3]. These data also have enormous potential to enable self-directed and personalized continuing professional development (CPD) and practice improvement for health professionals based on the data about their own clinical practice [4].

CPD is a cornerstone of health education and has a recognized place in maintaining high-quality care [5]. The principles of effective CPD have been well described in the literature and include being learner-centered, encompassing varied formats and delivery methods, and encompassing lifelong and ongoing assessments to address the needs of individual clinicians [6]. Another core component of health profession education is practice reflection by clinicians and clinical teams. Practice reflection describes the process of revisiting experiences both to learn from them and to understand the complex problems of professional practice [7]. Engaging in practice reflection is highlighted as being essential for health professionals to refresh and update their knowledge [8,9].

**Figure 1:**
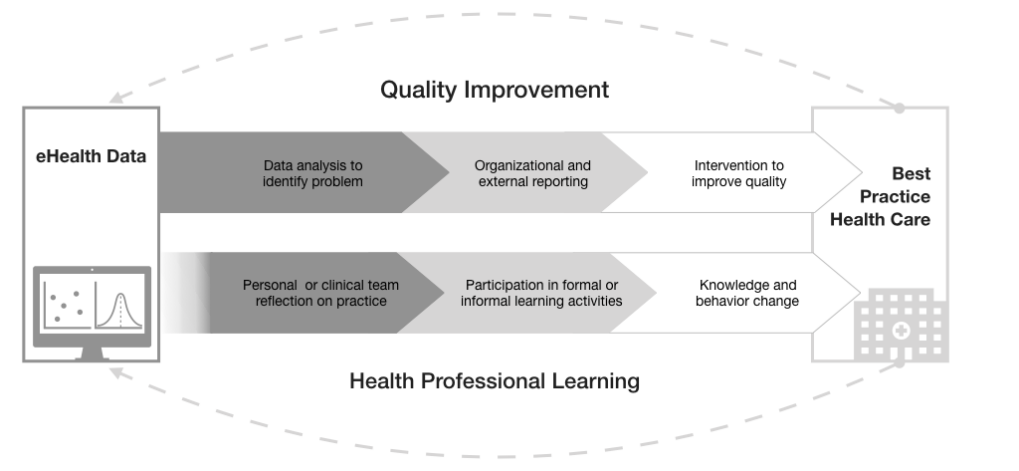
The difference between the use of eHealth data for quality improvement and health professional learning.

Although electronic data are a rich source of information for health professionals’ practice, health professionals currently have limited access to their data for this purpose [4]. eHealth data are used by quality improvement teams in many health care organizations to identify variations in the quality of care and lead the process of designing interventions to address this [2,10]. However, this process does not often provide health professionals with an opportunity to actively engage with eHealth data and have an active input in how the data are being used to improve practice. Figure 1 illustrates the disconnect between the processes by which eHealth data are used for quality improvement and how health professionals engage in learning activities.

Thus far, a notable gap in health professionals’ education and practice reflection is the use of eHealth data contained in clinical data sources such as electronic health records (EHRs), patient administrative systems, and registries. The value of using electronic data, such as that collected by EHRs for training and education, has been acknowledged in the literature [4]. Early research also suggests that clinicians themselves are keen to have more access to their data for personalized training and practice reflection [4]. The increasing need to incorporate clinical data into professional development and training has also been recognized at the government level in some countries [11]. In parallel to this, in some jurisdictions, the health care organization accreditation process now mandates audits and reviews of electronic data as part of the clinical governance process [12].

Despite the growing emphasis on the use of eHealth data to enable practice reflection, there are a number of challenges that need to be considered. A notable challenge relates to data management and ensuring the quality of data used while also considering privacy and security concerns [13]. Concerns about the quality of data collected by health informatics systems such as EHRs include the possibility that they may contain inaccurate or incomplete data, that the data are often recorded for coding purposes rather than quality improvement or other secondary applications, and that the data often do not indicate a complete clinical story [14]. A recent review of the literature on privacy and security of EHRs showed that health care organizations have a range of concerns regarding the security of data and, in response, implement a range of administrative, physical, and technical safeguards to help address them [15]. In addition, it is important to consider the views of consumers on the privacy and security of eHealth data. The literature on health information exchange has noted that barriers to consumers’ consent to health information exchange include privacy concerns, lack of awareness of the value of the contribution of their data, concerns about the data being used for profit, and the lack of easy tools for sharing their data [16,17].

Another challenge the health sector faces in harnessing the full potential of eHealth data is making the data actionable to health professionals in ways that can lead to improvements in quality, outcomes, or cost of care [13]. The application of digital technologies to facilitate better interactions between health professionals and electronic data for a range of professional development and quality improvement applications is a growing area in digital health [18-20]. The term Practice Analytics in Health care (PAH) is proposed to describe this area of digital health, which draws on key learnings from existing disciplines, including quality improvement, health professions education, health informatics, and learning analytics [21]. Broadly, PAH entails the use of integrated eHealth data sets to enable health professionals to optimize, improve, or enhance the value and quality of care. The field is heavily focused on identifying and collecting electronic data that surrounds individual health professionals and clinical teams, which can be used to obtain a snapshot of clinical practice, support individual learning, and enable learning systems. PAH harnesses data to generate new information and health insights that lead to practice reflection by clinicians and ultimately positive health outcomes. The field draws on 3 foundational academic disciplines: health and medicine, education and learning sciences, and engineering and information communication technology. Within these disciplines, there are a number of research specialties that contribute to PAH, including learning analytics [22], human–computer interaction [23], and quality improvement [24,25]. Textbox 1 provides a detailed overview of the research specialties that contribute to PAH.

**Overview of the foundational Practice Analytics in Health care research disciplines.**

**Academic disciplines and fields of research**

- Engineering and information communication technologies:
  Computer science
  Human–computer interaction
  Learning analytics
  Visualization
  Data analytics
  Machine learning
  Data mining
- Medicine and health:
  Epidemiology
  Public health
  Implementation science
  Quality improvement
  Ethics and law
  Data privacy and security
- Learning sciences and education:
  Medical education
  Health professions education
  Learning analytics

The program described in this paper seeks to understand how health professionals can be supported by digital technology to make effective use of eHealth data to support practice reflection. This will be achieved through a comprehensive research program delivered through a number of discrete doctoral research projects.

### Study Aims

The aim of this research program is to make eHealth data captured within tertiary health care organizations more actionable to health professionals for use in practice reflection, professional development, and other quality improvement activities, with the ultimate aim of improving patient outcomes.

## Methods

### Study Design

The research program described in this protocol is a problem-driven multidisciplinary research program between academic partners and the Digital Health Cooperative Research Centre (DHCRC), and industry partners made up of government and health service organizations. The research program is funded by the DHCRC [26]. Cooperative Research Centres are cofunded by the Australian government, industries, and universities to support applied research and development programs. In the context of this research program, a multidisciplinary team of researchers, scientists, and clinicians has been brought together to identify key industry problems and design a research program that addresses the abovementioned problems.

The multidisciplinary team guiding the delivery of the research program and supporting the project includes academic institutions (2/6, 33%), health care organizations (3/6, 50%), and peak bodies in the Australian health sector (1/6, 17%). The team was engaged early during the problem identification and research formulation stage, aligned with recommendations in the literature on how to undertake multidisciplinary research [27]. The research program will be delivered by a team of academic supervisors, doctoral candidates, and postdoctoral fellows working collaboratively to implement evidence-based solutions to the key problems and identify new knowledge that will address the research program’s aims. Supervisor panels were purposely drawn to represent academia, service partners, and active clinical practice.

The research program incorporates scientific theories from implementation science and action research. Action research is based on action, evaluation, and critical analysis of data to drive improvements, and it is commonly used to improve practices in various health care environments [28,29]. This type of research is facilitated by the participation and collaboration of a number of individuals with a common purpose. In PAH, action research is used to enable doctoral candidates to work collaboratively and undertake their research projects simultaneously while also reflecting and adapting based on each other’s learnings. Implementation science is the science of methods and strategies that facilitate the use of evidence-based research findings in regular practices by professionals and policy makers [30]. In this research program, implementation theory will be used to ensure that new knowledge created from the doctoral research projects is effectively transferred to tertiary hospitals to change behavior and alter clinical performance. The incorporation of these 2 theoretical approaches is central to ensuring that the program addresses both a research gap and meets the needs of industry partners.

### Research Program Development

The research program was co-designed by an academic and industry team supported by a program manager. Academic team members (n=5) brought expertise from a range of specialized research fields from the disciplines of medicine and health, engineering and information communication technology, and education and learning sciences. Industry team members (n=6) included representatives from private tertiary hospitals and representatives from peak national bodies.

To develop the study design, industry representatives who may have interest or expertise in using eHealth data with health professionals were identified from the DHCRC network. A one-on-one meeting with each industry representative and the lead investigator for the research program was conducted to identify key points and research priorities between May and July 2019. Meeting notes were synthesized into a preliminary research program outline by 2 researchers (TS and AJ) familiar with the area. This outline was then circulated to each industry representative who was interested in the research program for feedback.

Industry representatives who indicated continued interest in the direction of the research program participated in a planning workshop in August 2019. This workshop involved an open discussion between academic and industry attendees (n=14) to agree on the final objectives of the program. The workshop also identified the outline of the doctoral projects that would be achievable over the course of full-time candidature and that would meet the priorities of the industry representatives. Feedback from the planning workshop was reviewed and used to turn the research outline into a comprehensive research program road map. The research program was built around 3 streams. The protocol also described 6 objectives for the research program. Table 1 shows the objectives mapped against the 3 streams and the doctoral projects. The research program protocol was circulated to all workshop participants for consideration. Feedback on this document was incorporated into a revised version of the research program protocol.

**Table 1:** Overview of the 3 research streams in the Practice Analytics in Health care program.

Research stream and objectives		Doctoral project
**Stream 1: building capacity for the collection and use of meaningful eHealth data by health professionals**		
	Understand the readily available data that is most likely to be useful in performance feedback and continuous practice enhancement and explore the ethical considerations of using patient-reported outcomes and the barriers and enablers to health professionals using these data in practice	Defining clinical practice indicators Optimizing the actionability of patient-reported experience and outcome measures (future project)
**Stream 2: developing tools to optimize the use of complex health data by health professionals**		
	Determine the acceptability of different tools for feeding back performance data to individual clinicians and health care teams, understand the processes medical practitioners use to make sense of the data presented to them, and understand the ethical and policy implications for organizations and individual clinicians when using eHealth data for reflective practice	Visualizing performance data Data sensemaking Ethical, medico-legal, and policy implications of Practice Analytics (future project)
**Stream 3: understanding how Practice Analytics changes the behavior of health professionals and links with professional development**		
	Explore how performance data can be linked to professional development requirements, clinical governance, and hospital accreditation standards and understand how transition points in clinical careers influence the quality and usability of electronic data for practice reflection	Understanding the role of performance data in formal and informal professional education Exploring lifelong learning and career transition points in Practice Analytics (future project)

A final full-day workshop was held with academic and industry representatives (n=17) in February 2020 to review the final scope of the research program and confirm the doctoral projects and other logistical considerations. On the basis of the conclusion of the second workshop, there was a consensus on the 3 streams that would make up the research program. The scope for each of the 4 initial doctoral projects that would be embedded in the research program was also confirmed along with a road map for 3 future doctoral projects.

### Procedures

The research program will be enabled via an integrated set of doctoral research projects over a 5-year period. Ensuring the privacy and confidentiality of data is a key consideration for the research program. The research program procedures are in place with considerations for the research data being collected and the health data being used. To ensure that all research data are managed appropriately, each doctoral candidate will obtain appropriate human research ethics committee approval, which will require compliance with the National Statement on Ethical Conduct in Human Research [31]. In addition to research data, some projects in the research program may use health data. There are 2 types of health data that will need to be considered for the research program: patient data collected routinely by health care organizations and data about the professionalism of health professionals being used to develop practice reflection tools. It is not anticipated that identifiable patient data will be used in any of the research projects described in this protocol.

Regarding data about health professionals, individual doctoral candidates may use identifiable data. However, the process for accessing and using these data for each doctoral candidate will be approved by the relevant human research ethics committee. No identifiable data will be extracted or used without prior written consent. Finally, because of the importance of data privacy and ethical and legal considerations in the PAH space, the research program has prioritized a specific doctoral project to research these issues (Table 1).

**Figure 2:**
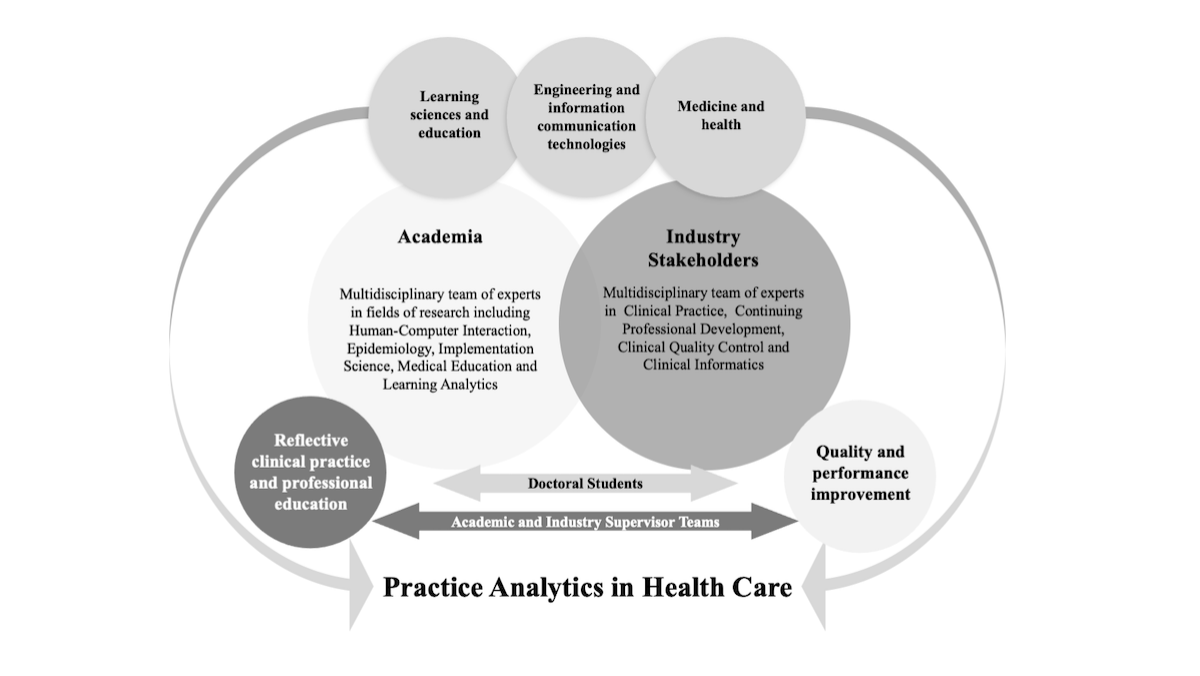
The collaborative methodology used to develop the Practice Analytics in Health Care research program.

Each doctoral research project is supported by a team comprising, at minimum, a primary academic supervisor with expertise in the research area, an industry supervisor with clinical or technological domain knowledge, and a postdoctoral fellow funded through the program. Figure 2 presents how inputs from academia and industry are harnessed to both design and deliver the Practice Analytics research program.

## Results

### Research Program Design

The process used to develop the research program identified 7 doctoral research projects to answer the program objectives, split across 3 streams. The following section presents a brief description of each doctoral research project and shows their alignment with the 3 streams of the research program. Table 1 shows the objectives mapped against the 3 streams and the doctoral projects.

### Stream 1: Building Capacity for the Collection and Use of Meaningful eHealth Data by Health Professionals

#### Defining Clinical Practice Indicators

This research project is designed to understand how to extract and structure data within health care organizations so that it can be used effectively for performance feedback. It is currently challenging to use eHealth data collected in health care organizations, such as data from patient administrative systems and electronic medical record (EMR), as the systems were not designed to capture data for this purpose. Currently, these systems are not designed with a primary focus on supporting health professional training and practice reflection. As such, it is necessary to explore how to repurpose these data for this project. The project will do this by identifying clinical indicators for individual diagnoses or procedures across different specialties that have clinical validity for the performance profiling of individual clinicians. Once the clinical indicators have been agreed upon by key stakeholders, a data audit will be undertaken to determine whether the quality and completeness of data within an individual health care organization are sufficient to populate each indicator by using routinely collected data.

#### Optimizing the Actionability of Patient-Reported Experience and Outcome Measures (Future Doctoral Project)

The project will explore the value of a generic set of patient-reported experience and outcome measures to use in informing clinical practice across key disciplines. The project will also explore the value of discipline-specific patient-reported experience and outcome measures and patient survey data sets for informing clinical practice.

### Stream 2: Exploring Different Approaches to Make Complex Health Data Actionable for Health Professionals

#### Visualizing Performance Data

This research project will explore how performance feedback tools are designed to enable health professionals to engage with their data. Research on the design of these platforms for use by health professionals is currently limited. This research project will address this problem by exploring the extent to which a one-size-fits-all solution is viable for feeding back performance data and contrasting it to more personalized approaches. When designing performance feedback tools, personalized approaches to visualizing data could account for factors such as health professional data literacy, different information needs, history of use of the data, and context-specific data use. It will also explore how performance feedback tools can support health professionals who desire a high level of engagement with their data rather than just supporting entry-level needs.

#### Sensemaking of Data

The overarching aim of the project is to understand how health professionals make sense of data related to their performance. Specifically, the project seeks to investigate how EMR and other health administration data are currently used by health professionals across different health care organizations. Currently, there is little research exploring the processes that health professionals use to make sense of their data, which is a problem because it is a barrier to the more widespread use of electronic data by this cohort. This project will address this problem by exploring the process of data sensemaking across different disciplines and organizational contexts. The project also aims to understand the factors that inform health professionals make sense of information such as emotion and previous experiences. Finally, the project will study how sensemaking informs further action in terms of both CPD and practice.

#### Ethical, Medico-Legal, and Policy Implications of Practice Analytics (Future Doctoral Project)

This research project is designed to understand the ethical and policy implications for organizations and individual clinicians when using eHealth data for reflective practice. The project will likely explore questions such as how organizations, individuals, and teams respond when data show an individual outlier that needs support and what information must be disclosed outside the organizations. The project will explore the way ethico-legal implications of using data for reflective practice affect engagement by specialists and other health professionals. This includes the nexus between using performance data from performance improvement rather than performance management and in potential litigation.

### Stream 3: Understanding How Practice Analytics Changes the Behavior of Health Professionals and Links With Professional Development

#### Understanding the Role of Performance Data in Formal and Informal Professional Education

This research project seeks to understand how medical regulatory bodies, education providers, and health care organizations can use eHealth data to personalize training programs. This is a challenge for health professionals, medical regulatory bodies, education providers, and other key stakeholders because there is an increasing expectation that health professionals will engage with their practice data for lifelong learning and other professional development activities. The project will address this problem by exploring how eHealth data from health care organizations can be used to better understand the clinical and professional practices of health professionals. Finally, the project will also investigate the attitudes of health professionals to the use of their data for professional development and reflective practice.

#### Exploring Lifelong Learning and Career Transition Points in Practice Analytics (Future Doctoral Project)

This research project is designed to understand how transition points in clinical careers influence the quality and usability of electronic data for practice reflection. The project will look specifically at the experiences of health professionals as they transition from specialist training to early-career specialty fellowships. It will also look at how data are presented and reported to health professionals in a way that captures knowledge, skills, and professional growth that occurs throughout a clinical career.

## Discussion

### Overview

This protocol paper provides a description of the term PAH and describes the academic disciplines that contribute to this multidisciplinary field. Furthermore, it presents an overview of the methodology for designing a PAH research program. The research program is the first to bring together academic disciplines combined with industry partners to identify and solve problems they face, increasing the actionability of eHealth data by health professionals for reflective practice and improvement. Finally, the protocol paper outlines how the aims of the research program will be achieved over the course of the program through an intervention that interlinked doctoral projects undertaken by doctoral candidates with diverse skill sets. At the conclusion of the research program, the intervention will have fostered strong partnerships between academic and industry partners; developed new knowledge in the field of PAH to address gaps in the research regarding the extraction, integration, and use of electronic data to change behavior; and translated key findings from the research program to clinical and regulatory settings to change practice.

A problem-driven multidisciplinary approach was foundational in designing the research program described in this protocol. To develop the PAH research program, experts with different perspectives and expertise worked collaboratively to refine the problem to focus on how electronic data within health care organizations could be used to support individual learning and strengthen professional development. The important role of multidisciplinary approaches involving academic and industry experts throughout the process can be challenging; however, through a guided process of discovery, ideation, and development, it can give voice to both health professionals and academic specialists to drive necessary changes [32]. In the area of PAH, multidisciplinary research is essential, as the field incorporates methodologies and approaches from diverse disciplines such as medicine and health, engineering and information communication technology, and learning sciences and education.

A key component of the research program is harnessing individual research projects to deliver an intervention that meets the needs of both academic and industry stakeholders. Each individual doctoral candidate brings knowledge and skills from their own discipline and applies them to answer a specific research question in service of the aims of the Practice Analytics research program. By harnessing principles from action research [28], the doctoral research projects run parallel in a complementary manner. Individual doctoral candidates meet regularly with each other and the larger program team to share their learnings and potentially refine their focus as the needs of the research program evolve. Individual projects are also embedded with industry partners to enable the health care system to absorb new knowledge from the research. Using discrete research projects that can run independently while also aligning with an overarching program has been established as an effective way of delivering multidisciplinary research [27].

To ensure that individual research projects are aligned with the goals of the PAH program, a number of processes have been implemented. These processes include having touch points with representatives of all industry partners supporting the Practice Analytics program, regular meetings between the academic supervisor team for each candidate, regular meetings between the academic supervisor team and the doctoral candidates, and regular meetings between the doctoral candidates and the postdoctoral fellows. These meetings are augmented with shared web-based repositories of information and serve the dual purpose of acting as coordination tools for the project and facilitating shared communication between all parties. Establishing an overall program management and coordination strategy is a recommended practice for successful multidisciplinary research programs [27].

This research program will need to draw on key learnings from different disciplines to address problems in the PAH field. An example of how this research program leverages research knowledge from diverse disciplines relates to a key challenge in the health sector: making eHealth data more actionable. To date, the health care industry has not fully achieved the potential benefits of using eHealth data to support health professional learning [4]. However, there is acknowledgment in the literature that using cyclic analysis of eHealth data should be incorporated into exemplary health learning environments [33]. Electronic data within health care organizations have not been widely used to support health professional learning for many reasons [34]; however, an important consideration is increasingly that one of the main mechanisms for capturing electronic data is EMRs. Most EMR systems were designed to create a digitized record of an individual’s data that can be used by health professionals to provide care to those individuals [35,36] and have limited functionality built into their design to support learning. Although this challenge is relatively new in the discipline of health and medicine, it has something that has been explored in the research area of human-computer interaction within the discipline of engineering and information communication technologies. Human-computer interaction specialists have identified that one of the barriers to developing good interfaces is that the system that is to be used was designed for a different purpose and its function has changed. By translating this knowledge from one discipline to another and between academia and industry, PAH has the potential to rapidly solve a range of problems faced by the health care industry.

Finally, because of the multidisciplinary nature of the research program, it is anticipated that outputs will have an effect not only on health care but also across a range of sectors. The research program will also incorporate established theories from implementation science [30] to ensure that new research knowledge is translated into practice change within the health care sector. At the conclusion of the program, it is anticipated that the intervention will create new knowledge to grow the field of PAH. Furthermore, this new knowledge will be translated into the health care sector within individual organizations to support health care professionals and teams and by regulatory bodies to personalize and strengthen professional development.

### Conclusions

The use of a multidisciplinary research program built around core objectives that align with the priorities of both industry and academic stakeholders is a unique approach that aims to unpack the potential of eHealth data to support learning, practice reflection, and other training activities of health professionals, improving the quality of care and patient outcomes. It is anticipated that this program will be extended to specific translational outputs informed by the research findings.

## Abbreviations

CPD: continuing professional development
DHCRC: Digital Health Cooperative Research Centre
EHR: electronic health record
EMR: electronic medical record
PAH: Practice Analytics in Health care
